# Tetrodotoxin Blockade on Canine Cardiac L-Type Ca^2+^ Channels Depends on pH and Redox Potential

**DOI:** 10.3390/md11062140

**Published:** 2013-06-14

**Authors:** Bence Hegyi, István Komáromi, Kornél Kistamás, Ferenc Ruzsnavszky, Krisztina Váczi, Balázs Horváth, János Magyar, Tamás Bányász, Péter P. Nánási, Norbert Szentandrássy

**Affiliations:** 1 Department of Physiology, University of Debrecen, P.O. Box 22, H-4012 Debrecen, Hungary; E-Mails: hegyi.bence@med.unideb.hu (B.H.); kistamas.kornel@med.unideb.hu (K.K.); ruzsnavszky.ferenc@med.unideb.hu (F.R.); vaczi.krisztina@med.unideb.hu (K.V.); horvath.balazs@med.unideb.hu (B.H.); magyar.janos@med.unideb.hu (J.M.); banyasz.tamas@med.unideb.hu (T.B.); szentandrassy.norbert@med.unideb.hu (N.S.); 2 Clinical Research Center and Thrombosis Haemostasis and Vascular Biology Research Group of the Hungarian Academy of Sciences, University of Debrecen, P.O. Box 40, H-4012 Debrecen, Hungary; E-Mail: komaromi@med.unideb.hu; 3 Department of Dental Physiology and Pharmacology, Medical and Health Science Center, University of Debrecen, P.O. Box 22, H-4012 Debrecen, Hungary

**Keywords:** tetrodotoxin, calcium current, dog heart, pH dependence, redox potential

## Abstract

Tetrodotoxin (TTX) is believed to be one of the most selective inhibitors of voltage-gated fast Na^+^ channels in excitable tissues. Recently, however, TTX has been shown to block L-type Ca^2+^ current (I_Ca_) in canine cardiac cells. In the present study, the TTX-sensitivity of I_Ca_ was studied in isolated canine ventricular myocytes as a function of (1) channel phosphorylation, (2) extracellular pH and (3) the redox potential of the bathing medium using the whole cell voltage clamp technique. Fifty-five micromoles of TTX (IC_50_ value obtained under physiological conditions) caused 60% ± 2% inhibition of I_Ca_ in acidic (pH = 6.4), while only a 26% ± 2% block in alkaline (pH = 8.4) milieu. Similarly, the same concentration of TTX induced 62% ± 6% suppression of I_Ca_ in a reductant milieu (containing glutathione + ascorbic acid + dithiothreitol, 1 mM each), in contrast to the 31% ± 3% blockade obtained in the presence of a strong oxidant (100 μM H_2_O_2_). Phosphorylation of the channel protein (induced by 3 μM forskolin) failed to modify the inhibiting potency of TTX; an IC_50_ value of 50 ± 4 μM was found in forskolin. The results are in a good accordance with the predictions of our model, indicating that TTX binds, in fact, to the selectivity filter of cardiac L-type Ca channels.

## 1. Introduction

It is generally believed that the marine guanidine toxin, tetrodotoxin (TTX), is a highly selective inhibitor of voltage-gated Na^+^ channels in various excitable tissues. This is really the case in skeletal muscle and neural tissues, where I_Na_ is blocked by TTX in the nanomolar range. In mammalian cardiac muscle, however, micromolar concentrations of TTX are required to suppress Na^+^ current effectively [1,2,3]; consequently, several tens of micromoles of TTX has to be applied in voltage clamp experiments when cardiac I_Na_ has to be eliminated.

TTX-sensitive Ca^2+^ current components have been identified in cardiac tissues under pathological conditions, such as in hypertrophied guinea pig or infarcted rat hearts [4,5]. TTX was shown to block L-type Ca^2+^ current in ventricular cardiomyocytes isolated from healthy dogs [6], *i.e.*, in a preparation having electrophysiological properties most similar to those of human ventricular myocardium regarding the distribution and kinetic properties of transmembrane ion currents [7,8].

In order to understand in detail how TTX blocks voltage gated Cav1.2 channels, structural information on their selectivity filter, derived from experimental atomic resolution 3D structures, is required. In absence of this information, a theoretical model to explain TTX binding to Cav1.2 channels was developed [6]. This model was based on the selectivity filter region of the Nav1.4 channel in complex with the TTX molecule and on the known homology between the Nav1.4, Nav1.5 and Cav1.2 channels [9,10]. Recently, the crystal structures of wild-type and mutated NavAb channels, including their most critical selectivity filter regions, have been published [11,12]. In their paper based on sequence alignment, Tikhonov and Zhorov concluded that the resolved X-ray structure of the point mutated bacterial NavAb channel can be a promising basis for modeling calcium channels [13].

In the present study, the TTX-sensitivity of I_Ca_ was re-examined in isolated canine ventricular myocytes as a function of changes in the extracellular pH, redox potential and channel phosphorylation in order to test our model experimentally. The results are congruent with the predictions of the model, suggesting that TTX binds, in fact, to the selectivity filter of the Cav1.2 channel.

## 2. Results and Discussion

### 2.1. Effect of Channel Phosphorylation

**Figure 1:**
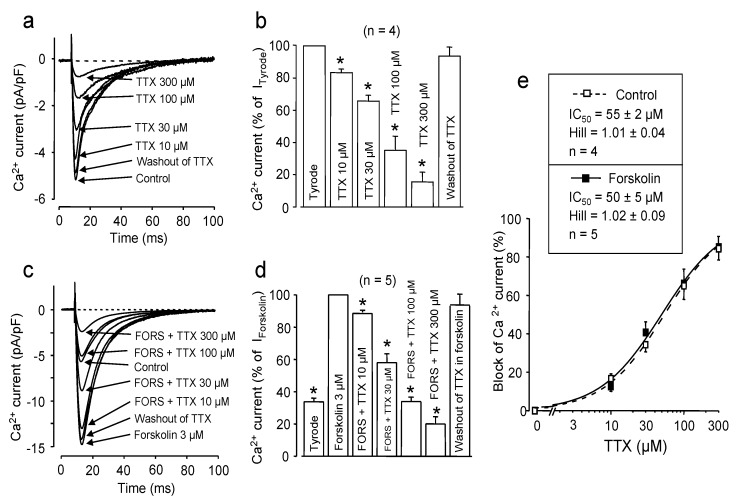
Effect of channel phosphorylation on the TTX-induced blockade. (**a**,**c**) Superimposed representative I_Ca_ records obtained in the presence of cumulatively increasing concentrations of TTX. Finally, TTX was washed out to demonstrate reversibility. Experiments were performed either in Tyrode solution (**a**) or in the presence of 3 μM forskolin (**c**). (**b**,**d**) Average results obtained with TTX in four untreated and five forskolin-treated myocytes (panels b and d, respectively). In both cases, peak I_Ca_ was normalized to the value measured prior to the first application of TTX. Columns and bars are the means ± SEM values; asterisks denote significant changes from the pre-TTX controls (Tyrode or forskolin, respectively). (**e**) Cumulative dose-response curves demonstrating the inhibitory effects of TTX in Tyrode solution (open symbols, dashed line) and in the presence of forskolin (filled symbols, solid line). IC_50_ values and Hill coefficients were determined by fitting data to the Hill equation.

Since phosphorylation of the channel protein is one of the most effective ways to control I_Ca_ in many excitable and non-excitable tissues, the blocking effect of TTX was compared on native and PKA-phosphorylated Ca channels. Forskolin is known to induce a stable activation of adenylate cyclase, resulting in high cAMP levels and, consequently, in full activation of protein kinase A, an enzyme responsible for phosphorylation of the pore forming α_1_ and the auxiliary β subunit of the Ca channel. This phosphorylation—through allosteric interactions—effectively increase the open probability of the pore-forming α1 channel subunit, while it has little effect on the selectivity filter [14]. As is demonstrated in Figure 1, 3 μM forskolin increased the peak amplitude of I_Ca_ (from 5.5 ± 0.4 pA/pF to 16.3 ± 0.8 pA/pF, *p* < 0.05, *n* = 5). TTX caused a concentration-dependent and reversible suppressive effect on I_Ca_ in both native and forskolin-treated cardiomyocytes. Furthermore, fitting the data to the Hill equation yielded very similar IC_50_ values: 55 ± 2 μM in control *vs.* 50 ± 5 μM in forskolin (*n* = 4 and *n* = 5, respectively, N.S.). The Hill coefficients in both cases were close to unity (1.01 ± 0.04 *vs.* 1.02 ± 0.09, N.S.). These results suggest that the phosphorylation-induced allosteric changes in the structure of the α1 subunit, although having serious consequences on channel gating, have little influence on TTX binding. The value of unity obtained for the Hill coefficients is congruent with the presence of one single TTX-binding site on each α_1_ subunit.

The 55 μM value of IC_50_ obtained with TTX for I_Ca_ is relatively high, even if considering that the cardiac Na channel isoform (Nav1.5) shows much lower affinity to TTX than the neural (Nav1.1–1.3) or skeletal (Nav1.4) ones [15,16,17]. The aromatic side chain of the residue following the DEKA ring builder Asp residue was proposed to play a critical role in the specific TTX-ion channel interaction, and its replacement (e.g., to Cys residue in Nav1.5) may be the main reason for the reduced affinity [15,16]. In the sequence alignment proposed by Tikhonov and Zhorov [13], which was used when building the model in the present study, a Gly residue can be found at this key position. This fact can plausibly explain why Cav1.2 channels bind TTX with considerably lower affinity than neural or skeletal Na^+^ channels.

### 2.2. Effects of Extracellular pH

**Figure 2:**
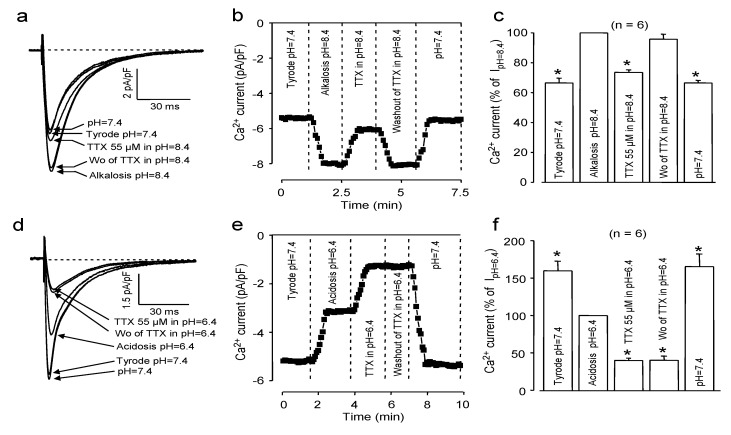
Effect of extracellular alkalosis (**a**–**c**) and acidosis (**d**–**f**) on the TTX-induced I_Ca_ blockade. Superimposed representative I_Ca_ records (**a**,**d**), time-dependent changes in peak I_Ca_ (**b**,**e**) and average values of peak I_Ca_ (**c**,**f**). Data were first obtained in Tyrode solution at pH = 7.4; then, the pH was shifted to either the alkaline or acidic direction (pH = 8.4 and pH = 6.4, respectively), and the cell was exposed to 55 μM TTX. Finally, TTX was removed, and in the next step, pH was turned back to its control value of 7.4. Columns and bars represent the means ± SEM; asterisks indicate significant changes from the pre-TTX controls (alkalosis or acidosis, respectively).

Our model, constructed to explain the TTX-sensitivity of I_Ca_, predicts that TTX binds to the selectivity filter of the channel [6]. More specifically, this connection is based on the negatively charged (at pH values close to the physiological range) selectivity filter region of the channel and the positively charged guanidine group of the toxin. Consequently, changing the strengths of this electrostatic interaction by increasing or decreasing the protonation of either the selectivity filter region of the channel or of the TTX molecule itself is expected to modulate the magnitude of the TTX-induced blockade. As demonstrated in Figure 2, alkalization significantly decreased (to 26% ± 2% at pH = 8.4, *n* = 6), while acidification increased (to 60% ± 2% at pH = 6.4, *n* = 6) the inhibition of I_Ca_ caused by 55 μM TTX (which was estimated to be exactly 50% at the physiological pH value of 7.4).

It is also worthy of noting that the effect of TTX could not be reverted at acidic pH, suggesting that the toxin was bound strongly under these conditions. Indeed, TTX could be removed from the channel, resulting in a full reversion of blockade, when pH was finally restored to 7.4 in TTX-free Tyrode solution (see Figure 2e,f).

Regarding the effect of pH on the amplitude of I_Ca_, a very simplified interpretation can be given. Considering the approximately 6.5 pKa value of histidine [18], its imidazole side chain can be protonated, even in a mild acidic milieu. It should be noted, however, that the actual pKa depends strongly on the chemical environment. Accordingly, the histidine residues located in the conduction pathway of the channel (including the selectivity filter, as well) are at least partially protonated at the acidic pH of 6.4, which resulted in a less favorable environment for a positively charged ion. In addition, one (or even more than one) of the glutamate side chains at the highly negatively charged EEEE ring of the channel can be protonated, too, which can further reduce cationic current. Indeed, similarly to our results, I_Ca_ and I_Na_ have been reported to decrease in acidic milieu [15,16,19,20]. On the other hand, TTX exists almost exclusively in its positively charged form at pH 6.4, since the pKa value of the most acidic C10 hydroxyl group is close to 8.7 [21]. Therefore, the positively charged TTX molecule is expected to increase TTX binding (compared to the neutral zwitterionic form, which is dominant at the higher, alkaline pH range, but also existing partially at pH = 8.4 and in a small fraction even at pH = 7.4) to the negatively charged selectivity filter region resulting in a stronger inhibition of I_Ca_. Furthermore, due to the reduced strength of electrostatic interactions (which is valid for both the double charged Ca^2+^ and—although to a lesser extent—for the protonated TTX molecule, because of its single positive charge), the relative contribution of the non-ionic hydrophilic and hydrophobic terms in the interaction between TTX and the selectivity filter of the channel is likely to increase. All these effects resulted in a reduction of I_Ca_ in acidic environment, which is still strongly inhibited by TTX. In contrast, at the alkaline pH of 8.4, most of the histidine side chains are neutral, and largely half of the free cysteine SH groups can be deprotonated, since the corresponding pKa is 8.5 [18]. This is certainly a more favorable environment (even compared to the neutral situation, *i.e.*, to the case of pH = 7.4) for attracting and binding of the positively charged Ca^2+^ ions to the channel; consequently, the ion flux through the channel may increase. TTX may exist partially in its neutral form at mild alkaline pH values, due to deprotonation of the C-10 hydroxyl group, allowing for development of a weaker electrostatic attraction between TTX and the selectivity filter. The stronger Ca^2+^ channel interaction combined with a weaker interaction between TTX and the channel protein necessarily yields a smaller inhibition of I_Ca_ by TTX under alkaline conditions.

The increased amplitude of I_Ca_ and the decreased TTX blockade, observed at alkaline milieu, can easily be interpreted based on the simplified electrostatic model and the protonation equilibrium of the zwitterionic TTX outlined above in good agreement with the pH dependence of the TTX blockade observed on Na channels [22,23]. The increased inhibitory effect of TTX at acidic pH seems to contradict earlier results, suggesting a reduced TTX block at low pH values [22]. The reason for this discrepancy is not fully understood; however, one should consider that the structure of Ca and Na channels differs due to the limited sequence identity, and even in the case of Na channels, the corresponding effect is not especially large at a neutral or slightly acidic pH range. It is possible that at pH = 6.4, the conformation of the selectivity filter region of the Ca channel may change in a way, becoming more suitable for TTX-binding. Moreover, the 3%–5% non-effective (zwitterionic) fraction of TTX, estimated at pH = 7.4, is converted into cationic form at pH = 6.4, which may also contribute to the increase of the TTX-induced block. 

### 2.3. Effects of Redox Potential Changes

**Figure 3:**
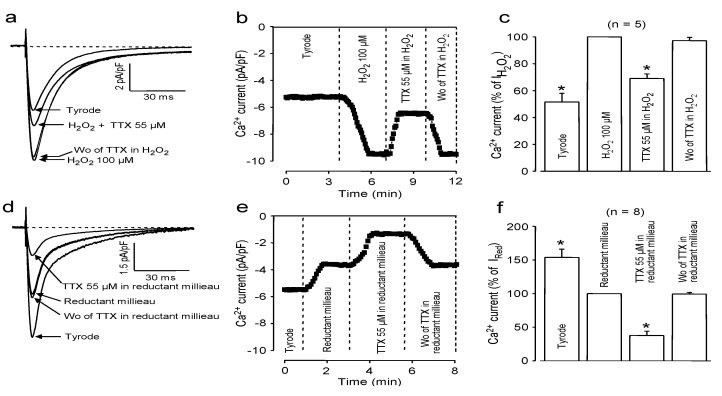
Effect of oxidant (**a**–**c**) and reductant (**d**–**f**) external milieu on the TTX-induced I_Ca_ blockade. Superimposed representative I_Ca_ records (**a**,**d**), time-dependent changes in peak I_Ca_ (**b**,**e**) and average values of peak I_Ca_ (**c**,**f**). Data were first obtained in Tyrode solution; then, the superfusate was made either oxidant (by 100 μM H_2_O_2_) or reductant (by application of glutathione + ascorbic acid + dithiothreitol, 1 mM each). The cells were exposed to 55 μM TTX in these solutions, followed by washout of TTX. Columns and bars represent the means ± SEM; asterisks indicate significant changes from the pre-TTX controls (H_2_O_2_ or reductant milieu, respectively).

As demonstrated in Figure 3, the effects of redox potential changes were very similar to those initiated by changes in pH. Accordingly, in a strongly oxidant milieu, induced by application of 100 μM H_2_O_2_, 55 μM TTX suppressed only 31% ± 3% of I_Ca_ (Figure 3a–c, *n* = 5), in contrast to the 62% ± 6% I_Ca_ blockade observed in a reductant environment (Figure 3d–f, *n* = 8, 1 mM reduced glutathione + 1 mM ascorbic acid + 1 mM dithiothreitol). The role of the redox potential in modulating I_Ca_ and its suppressibility by TTX needs some more effort to be interpreted. In line with our observations, hydrogen peroxide has been shown to increase the amplitude of I_Ca_ in various mammalian cardiac tissues [24,25,26], while the current was decreased by a reductant environment [27]. Since H_2_O_2_ is a light oxidant, it oxidizes the free sulfhydryl groups to disulfide bonds. In contrast, a reducing environment may prevent the formation of disulfide bridges or break them once already performed. These disulfide bridges seem to stabilize a structure relatively resistant to TTX binding; thus, the inhibitory action of TTX on I_Ca_ increases in a reductant environment, while it decreases in the presence of H_2_O_2_. An additional explanation arises when considering that the density of I_Ca_ correlates well with oligomerization of the Cavβ subunits of Ca channels [28]. Assuming that H_2_O_2_ can stabilize these oligomeric states by the formation of disulfide bridges, both the higher I_Ca_ amplitudes, as well as the weaker inhibition by TTX can be explained.

Finally, it must be noted that the effects of a reductant milieu, both on the amplitude of I_Ca_ and on its suppressibility by TTX, were very similar to those of acidification. This strongly suggests that application of a reducing milieu may facilitate the protonation of some sensitive groups—in spite of the large buffering capacity of the system. This might explain the good coincidence observed in the TTX-induced inhibition of I_Ca_, comparing it in an acidic *vs.* alkaline and in a reductant *vs.* oxidant milieu.

### 2.4. Use-Dependent Block

**Figure 4:**
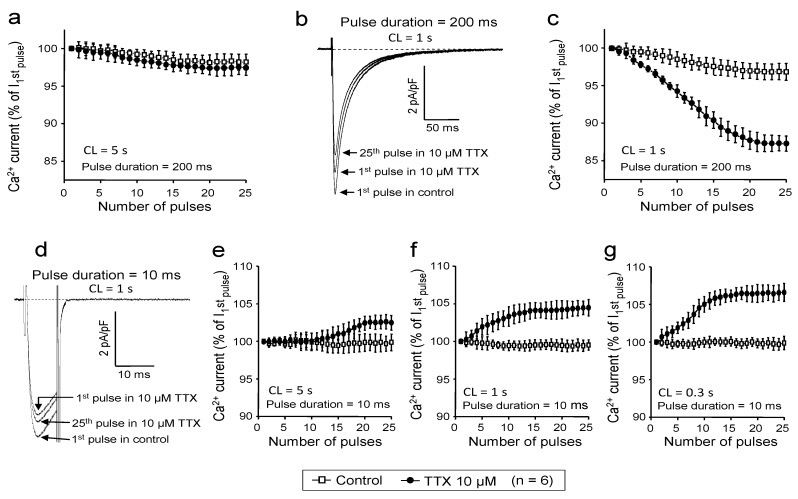
Rate-dependent effects of TTX on I_Ca_. Stimulating voltage pulses were applied at various cycle lengths, as indicated in each panel, following a 1 min period of rest (stimulation-free period). Peak values of I_Ca_, obtained during the initial 25 pulses were plotted in the absence (open squares) and presence (filled circles) of 10 μM TTX. I_Ca_ was expressed as a percent of current amplitude observed during the first pulse. Pulse duration was set to either 200 ms (**a**–**c**) or 10 ms (**d**–**g**). Superimposed representative I_Ca_ recordings, obtained using 200 ms and 10 ms depolarizations at 1 Hz, are displayed in panels **b** and **d**, respectively.

In the experiments above, I_Ca_ was activated by 400 ms-long depolarizations delivered at a frequency of 0.2 Hz. In other words, the repetition time of stimulation was 5 s. This was long enough to maintain a reasonably normal availability of I_Ca_; therefore, the effect of TTX observed under these conditions was considered as the magnitude of tonic block. Indeed, as demonstrated in Figure 4a, this slow rate resulted in only a small reduction in I_Ca_ amplitude during the first 25 pulses applied following a 1 min period of rest both in the absence and presence of TTX (2.0 ± 1.0 and 2.8% ± 1.1% reduction, respectively, N.S., *n* = 6). However, a significant rate-dependent component of block was built up during the initial 25 pulses in the presence of 10 μM TTX, when the cycle length of stimulation was increased to 1 s. The magnitude of this rate-dependent block was 12.7% ± 1.0% *vs.* the 4.3% ± 1.1% decrease of I_Ca_ observed without TTX (Figure 4b,c, *p* < 0.05, *n* = 6). The 200 ms duration of these pulses did not allow the studying of the effect of TTX at higher frequencies; therefore, short depolarizations, having durations of only 10 ms, were applied in control and in the presence of TTX. Surprisingly, these short pulses caused a rate-dependent enhancement of I_Ca_ in the presence of 10 μM TTX, but not in control (Figure 4d–g). Increases in I_Ca_ amplitudes were larger at higher stimulation rates: increases of 2.1% ± 1.1%, 3.9% ± 1.2% and 6.3% ± 1.2% were obtained at cycle lengths of 5 s, 1 s and 0.3 s, respectively. These results indicate that in addition to the tonic block, an additional rate-dependent block is also evoked by TTX, however, the magnitude of this block was relatively moderate compared to the tonic component. More importantly, the TTX-induced inhibition of I_Ca_ seems to be restricted to resting and inactivated channel states, since frequent opening of the channel (by using short depolarizations at high frequencies) results in a relief from the block, suggesting that the affinity of TTX to the open channel state is markedly reduced.

### 2.5. Simulations

**Figure 5:**
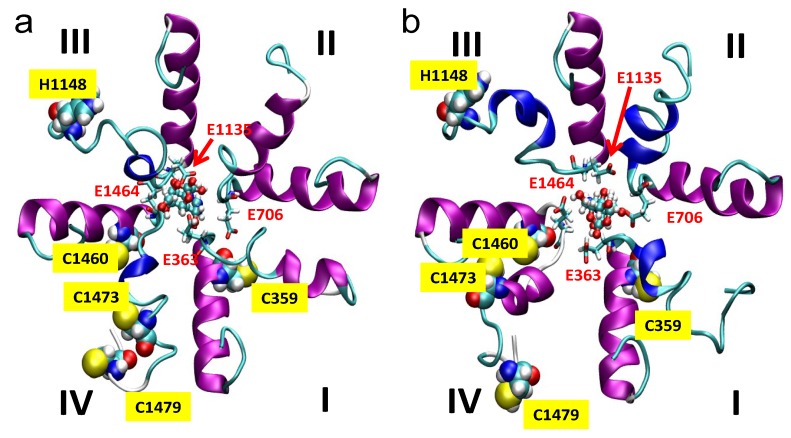
Representative structure snapshots from dynamic trajectories of the selectivity filter region of the L-type Ca^2+^ channel with TTX being in a positively charged form (**a**), with protonated guanidine and the non-dissociated C10–OH group, and in neutral form (**b**), with protonated guanidine and the proton dissociated from the C10-OH group resulting in C10–O^−^. The repeats are marked by Roman numerals. The EEEE ring of the selectivity filter and the TTX molecule are shown using “liquorice” and ball-and-stick representation, respectively. The position of the His and Cys residues marked by the van der Waals spheres of the atoms constitute these residues. The residue numbering is based on the human L-type Cav1.2 channel sequence obtained from the UNIPROT protein knowledgebase.

Since the pKa value (8.7) of the most acidic OH in the TTX molecule is relatively close to the pH range of 6.4–8.4 applied in our experiments, both the protonated form (*i.e.*, positively charged due to the guanidinium group and the C10–OH) and the neutral form (the guanidinium group of TTX is still protonated, but the C10–OH is deprotonated to C10–O^−^) of TTX were considered in our calculations (Figure 5).

Docking experiments revealed that TTX binds to the selectivity filter in both of its states; however, the binding is stronger (8.22 kcal/mol *vs.* 7.55 kcal/mol) when TTX is positively charged (*i.e.*, when the proton is not dissociated from the C10–OH position). This can be the consequence of the dominantly negative formal charge of the selectivity filter. Molecular dynamics simulation started from the docked conformations supported the results obtained by docking, as the complexes remained stable during the 65 ns period of simulation (see the movies in the Supplementary Material).

## 3. Experimental Section

### 3.1. Isolation of Single Canine Ventricular Myocytes

Adult beagle dogs of either sex were anaesthetized with intravenous injections of 10 mg/kg ketamine hydrochloride (Calypsol, Richter Gedeon, Budapest, Hungary) + 1 mg/kg xylazine hydrochloride (Sedaxylan, Eurovet Animal Health BV, Bladel, The Netherlands), according to a protocol approved by the local ethical committee and conforming to “principles of laboratory animal care” (NIH publication No. 85–23, revised 1985). The hearts were quickly removed and placed in Tyrode solution. Single myocytes were obtained by enzymatic dispersion using the segment perfusion technique [29,30]. Briefly, a wedge-shaped section of the ventricular wall supplied by the left anterior descending coronary artery was dissected, cannulated and perfused with oxygenized Tyrode solution containing (in mM): NaCl, 144; KCl, 5.6; CaCl_2_, 2.5; MgCl_2_, 1.2; HEPES, 5; dextrose, 11; at pH = 7.4. Perfusion was maintained until the removal of blood from the coronary system and, then, switched to a nominally Ca^2+^-free Joklik solution (Minimum Essential Medium Eagle, Joklik Modification, Sigma-Aldrich Co., St. Louis, MO, USA) for 5 min. This was followed by 30 min perfusion with Joklik solution supplemented with 1 mg/mL collagenase (Type II., Worthington Biochemical Co., Lakewood, NJ, USA) and 0.2% bovine serum albumin (Fraction V., Sigma-Aldrich Co., St. Louis, MO, USA) containing 50 μM Ca^2+^. Portions of the left ventricular wall were cut into small pieces, and the cell suspension, obtained at the end of the procedure predominantly from the mid-myocardial region of the left ventricle, was washed with Joklik solution. Finally, the Ca^2+^ concentration was gradually restored to 2.5 mM. The cells were stored in minimum essential medium until use.

### 3.2. Electrophysiology

All measurements were performed at 37 °C. The rod-shaped viable cells showing clear striation were sedimented in a Plexiglas chamber allowing continuous superfusion with oxygenized Tyrode solution. Suction pipettes, fabricated from borosilicate glass, had a tip resistance of 2 MΩ after filling with pipette solution containing in (mM): KCl, 110; KOH, 40; HEPES, 10; EGTA, 10; TEACl, 20; K-ATP, 3 mM, at pH = 7.2. I_Ca_ was recorded in Tyrode solution supplemented with 3 mM 4-aminopyridine with Axopatch-2B amplifier (Axon Instruments Inc., Foster City, CA, USA) using the whole cell configuration of the patch clamp technique [31]. The current was activated at +5 mV using 400 ms-long depolarizations arising from the holding potential of −40 mV. After establishing a high (1–10 GΩ) resistance seal by gentle suction, the cell membrane beneath the tip of the electrode was disrupted by further suction or by applying 1.5 V electrical pulses for 1 ms. The series resistance was typically 4–8 MΩ before compensation (usually 50%–80%). Experiments were discarded when the series resistance was high or substantially increasing during the measurement. Outputs from the clamp amplifier were digitized at 100 kHz under software control (pClamp 6.0, Axon Instruments Inc., Foster City, CA, USA). Ion currents were normalized to cell capacitance, which was determined in each cell using short hyperpolarizing pulses from −10 mV to −20 mV.

### 3.3. Simulation of TTX Binding to Cav1.2 Channels

The selectivity filter region of the human Cav1.2 channel was built on the basis of the X-ray structure of the selectivity filter region of the bacterial NavAb channel [12] and the existence of homology between the NavAb and Cav1.2 channels. The alignment between NavAb and Cav1.2 has been previously published [13], which was used without modification. Proper position of the EEEE ring, *i.e.*, E363, E706, E1135 and E1464 residues in the human L-type Cav1.2 channel, confirmed this alignment [10]. The residue mutations corresponding to NavAb to Cav1.2 transformations were then carried out by means of the MODELER software [32] using the Chimera package as the graphical front-end [33]. The TTX molecule was then docked to the model using the Autodock4 software with the help of the graphical front-end “MGL tools” software suite [34]. In docking calculations, the flexible ligand (rotatable OH groups) rigid receptor model with genetic algorithm was applied [35]. Both the positively charged and the neutral (with deprotonated oxygen) form of TTX were docked to the selectivity filter of the Cav1.2 model. In order to assess the stability of the complexes, molecular dynamics simulations were carried out starting from the geometry obtained by docking. In the simulations for the selectivity filter and TTX molecule, AMBER99SB and GAFF force fields were applied, respectively [36,37]. Since only the selectivity filter region of Cav1.2 was included in the calculations, position restraints for the *N*- and *C*-terminal residues of each repeat-fragment and for the backbone atoms of the helical structures constituting the outer part of the filter region (*N*-terminal part of corresponding repeat-fragment) with no direct interaction with the TTX molecule were applied. All other atoms and residues were allowed to move freely during the simulation. The short constant particle number, constant pressure and constant temperature molecular dynamics (NPT) equilibration period was followed by the constant volume (NVT) simulation. In the calculations, explicit TIP3P water molecules and periodic boundary conditions were applied [38]. The long range electrostatic interactions were calculated using the particle mesh Ewald method [39]. Applying 2 fs time-steps, the total length of NPT and NVT simulations were 2 ns and 65 ns, respectively. The ionic strength was set to 150 mM by adding Na^+^ and Cl^−^ ions to the simulation box. The simulations were carried out using the Amber 12 software package [40], while for visualization and movie generation, the visual molecular dynamics (VMD) package was used [41].

### 3.4. Statistics

Results are expressed as the mean ± SEM values. Statistical significance of differences was evaluated using the Student’s *t*-test for paired or unpaired data, as pertinent. Differences were considered significant when *p* was less than 0.05. Drugs were obtained from Sigma-Aldrich Co. (St. Louis, MO, USA).

## 4. Conclusions

By applying acidic, alkaline, reductant and oxidant environments combined with *in silico* simulations, we have demonstrated in the present study that the guanidine-toxin TTX inhibits L-type Ca channels in the heart by binding to its selectivity filter—in a way similar to its action on fast Na channels [15,42].

## Supplementary Files

Supplementary File 1 Supplementary (MPG, 8807 KB)
